# Correlation Between the Prevalence of Temporomandibular Disorders and Their Association With Psychological Distress in Central Saudi Arabia

**DOI:** 10.7759/cureus.38462

**Published:** 2023-05-02

**Authors:** Faris Almutairi, Norah Alzamil, Othman Alkhuzayyim

**Affiliations:** 1 Department of Maxillofacial Surgery and Diagnostic Sciences, Qassim University, Qassim, SAU; 2 Medical School, Qassim University, Qassim, SAU

**Keywords:** psychological distress, dentistry, temporomandibular joint disorders, screener, anxiety, depression, pain

## Abstract

Background

In this study, we aimed to determine the prevalence of temporomandibular disorders (TMDs) and their association with psychological distress in the central region of Saudi Arabia.

Methodology

In this cross-sectional study, a questionnaire was sent randomly to residents of Al-Qassim province. They were asked to complete a TMD pain screener, the Patient Health Questionnaire-4 (PHQ-4), and the Generalized Anxiety Disorder Scale (GAD-7). Correlations between symptoms of pain-related TMDs and PHQ-4 and GAD-7 scores were analyzed using Spearman’s correlation test. Frequencies and percentages were calculated for sex, age, TMD, PHQ-4, GAD-7, and TMD pain-screener responses. A chi-square test was performed to determine the association between demographic data and psychological profiles.

Results

The majority of the respondents (59.4%) reported at least one symptom of pain-related TMDs. The TMD pain score was positively correlated with PHQ-4 and GAD-7 scores.

Conclusions

Residents of the Al-Qassim region who experienced elevated levels of psychological distress had significantly more pain-related TMD symptoms. These findings imply a link between psychological distress and TMD symptoms.

## Introduction

The pain associated with temporomandibular disorders (TMDs) is a common chronic pain condition [1] following dental pain [2,3]. Although the signs and symptoms of TMD, including those other than pain around the joint area (such as limited mouth opening and clicking joint sounds), are relatively high, relatively few patients seek medical advice for TMDs [1-4]. Pain is the main complaint when patients seek medical management [1-4]. The prevalence of TMDs has been linked to sociodemographic status in an increasing number of studies. A higher incidence of TMDs is found in individuals aged 18 to 44 years, and women are four times more likely to have TMDs than men [5,6]. Psychosocial symptoms often accompany chronic TMD pain, resulting in depression, disability, and decreased health and quality of life [7,8]. In both early and late stages of life, TMD pain is associated with negative psychosocial status [9]. Therefore, it is important to continually evaluate the psychosocial characteristics of TMD patients, as proposed in the Diagnostic Criteria for Temporomandibular Disorders [9,10]. One prospective cohort study revealed that TMD pain is roughly twice as likely to develop in patients with depression and anxiety [11]. A link between psychological distress, such as anxiety or depression, and myofascial pain was reported in a cross-sectional study [11]. However, no credible research has investigated depression and anxiety individually as risk factors for pain-related TMDs.

In this study, a TMD pain screener, the Patient Health Questionnaire-4 (PHQ-4), and the Generalized Anxiety Disorder Scale (GAD-7) were used to determine the prevalence of self-reported, pain-related TMDs among Al-Qassim region residents, to investigate the correlation between PHQ-4 and GAD-7 total scores (anxiety and depression subscale scores) and symptoms of pain-related TMDs, and to compare levels of pain-related TMD symptoms between subjects with different psychological profiles.

## Materials and methods

Study design and subjects

This study was approved by the Qassim ethical committee for research (approval number: 607-44-7578). In this cross-sectional study, a questionnaire assessing symptoms of pain-related TMDs and psychological distress was sent using convenience sampling for Al-Qassim residents in Saudi Arabia. Demographic data (e.g., sex and age) were also obtained. The required sample size was calculated based on previous related studies [12,13]. Based on the reported population of Al-Qassim, the sample size was calculated. A minimum of 175 participants was necessary with a 95% confidence interval and 80% power.

TMD pain evaluation

Participants responded to the six-question extended TMD pain screener, which assessed signs and symptoms of pain-related TMDs. Excellent sensitivity and specificity have been reported for this TMD pain screener for identifying pain-related TMDs [14]. The questionnaire items included the duration of pain, the presence of pain or stiffness in the jaw upon waking, and activities that affect pain, such as chewing hard food, jaw movements, and jaw habits. The score ranges from 0 to 7, with a score of at least 2 indicating pain-related TMDs. In addition, two more items were added to the TMD pain scanner. The first queried trouble sleeping as TMD patients frequently have insomnia or difficulty falling asleep as a result of TMJ pain. The second queried whether they sought treatment as seeking medical or dental care should be taken into account when gauging patient awareness.

Psychological evaluation

Each participant answered the PHQ-4, a valid and accurate screening instrument for psychological distress [15]. There are four questions on the survey. Scores for each response range from 0 (not at all) to 3 (almost daily). There are a total of 12 possible scores, with higher values suggesting higher intensity. A score of 2 suggests no or minimum depression or anxiety, a score of 3-5 mild depression or anxiety, a score of 6-8 moderate depression or anxiety, and a score of 9 severe depression or anxiety. The PHQ-4 can be used to assess depression and anxiety separately because it combines the PHQ-2 (a depression screener) and the GAD-7 (an anxiety screener). Participants were deemed to have depression or anxiety if they scored more than 3 on the PHQ-2 and GAD-7 subscales [15,16].

Data analysis

Data analysis was performed using the SPSS program for Windows version 22 (IBM Corp., Armonk, NY, USA). Frequencies and percentages were calculated for sex, age, TMD, PHQ-4, GAD-7, and TMD pain screener responses. The chi-square test was performed to determine the association between demographic data and psychological profiles. Spearman correlation was performed to determine the relationship between TMD score and PHQ-4 and GAD-7. Statistical significance was defined as p ≤0.05.

## Results

In total, 224 patients completed the survey with no missing data. The respondent distribution by demographic characteristics and frequency of each reported pain-related TMD symptom is presented in Table 1. Almost three-quarters of the respondents were females 167 (74.6%) with 57 males (25.4%). The majority of the respondents, 140 (62.5%), were aged 18-39 years; 58 (25.9%) respondents were aged 40-59 years; and 24 (10.7%) respondents were aged more than 60. Finally, only two participants were under 18 (0.9%), as shown in Table 1.

**Table 1:** Respondent distribution by demographic characteristics. TMD = temporomandibular disorder

Variable	N(%)
Sex	
Male	57 (25.4)
Female	167 (74.6)
Age (years)	
<18	2 (0.9)
18–39	140 (62.5)
40–59	58 (25.9)
>60	24 (10.7)
TMD	
Negative	151 (67.4)
Positive	73 (32.6)
TMD symptoms	
None	91 (40.6)
At least one symptom	133 (59.4)

Pain-related TMD was present in 73 (32.6%) respondents, whereas negative pain-related TMD was noted in 151 (67.4%) respondents. The majority of the respondents, 133 (59.4%), reported at least one symptom of pain-related TMDs, whereas 91 (40.6%) showed no symptoms whatsoever (Table 1).

The PHQ-4 scores showed that 95 (42.4%) respondents scored normal and 85 (37.9%) scored mild. Additionally, 25 (11.2%) respondents scored moderate, and 19 (8.5%) respondents scored severe. As for the GAD-7, 93 (41.5%) respondents scored minimal, and 76 (33.9%) scored mild. Interestingly, GAD-7 showed an increase in moderate, 32 (14.3%), and severe, 23 (10.3%), scores (Table 2).

**Table 2:** PHQ-4 and GAD-7 scores. PHQ-4 = Patient Health Questionnaire-4; GAD-7 = Generalized Anxiety Disorder Scale

Variable	N (%)
PHQ-4	
Normal	95 (42.4)
Mild	85 (37.9)
Moderate	25 (11.2)
Severe	19 (8.5)
GAD-7	
Minimal	93 (41.5)
Mild	76 (33.9)
Moderate	32 (14.3)
Severe	23 (10.3)

In total, 27 (37%) respondents reported having trouble sleeping while having TMD-positive scores, and 46 (63%) respondents had no trouble sleeping. Regarding seeking medical or dental treatment, only seven (9.6%) respondents had gone to a physician, and the majority, 66 (90.4%), had not sought medical or dental treatment (Table 3).

**Table 3:** Questions related to TMD-positive patients and TMD pain screener questions. TMD = temporomandibular disorder

Variable	N (%)
Question-related to TMD-positive patients (n = 73)	
Did you have trouble sleeping?	
No	46 (63)
Yes	27 (37)
Did you seek medical or dental treatment?	
No	66 (90.4)
Yes	7 (9.6)
TMD pain screener questions (n = 224)	
In the last 30 days, on average, how long did any pain in your jaw or temple area on either side last?	
No pain	149 (66.5)
Pain comes and goes	66 (29.5)
Pain is continuous	9 (4)
In the last 30 days, have you had pain or stiffness in your jaw on awakening?	
No	184 (82.1)
Yes	40 (17.9)
In the last 30 days, did the following activities change any pain (that is, make it better or make it worse) in your jaw or temple area on either side?	
Chewing hard or tough food	40 (17.9)
Jaw habits such as holding teeth together, clenching, grinding, or chewing gum	47 (21)
Opening your mouth or moving your jaw forward or to the side	19 (8.5)
Other jaw activities such as talking, kissing, or yawning	10 (4.5)
None of the above activities change the pain	108 (48.2)

On the TMD pain screener questionnaire, 149 (66.5%) had no pain in the past 30 days in their jaw or temple area on either side, whereas nine (4%) respondents had continuous pain, and 66 (29.5%) respondents had intermittent pain. Additionally, 184 (82.1%) participants did not have pain or stiffness in their jaw on awakening, whereas 40 (17.9%) had pain (Table 3).

Table 4 shows that sex and TMD were dependent, where 61 (83.6%) TMD patients were female and only 12 (16.4%) were male (p = 0.021). Age and TMD were independent, where the p-value was 0.763, greater than 0.05. There was a significant relationship between PHQ-4 and TMD, where 10 (13.7%) respondents with a TMD had a severe PHQ-4 score compared to nine (6%) non-TMD respondents (p = 0.001). Finally, there was a significant relationship between GAD-7 and TMD, where 10 (13.7%) participants with TMD had severe GAD-7, and 20 (27.4%) had minimal GAD-7. Additionally, 73 (48.3%) non-TMD respondents had a minimal score on the GAD-7 (Table 4).

**Table 4:** Comparison of sex, age, PHQ-4, and GAD-7 scores between TMD-positive and negative patients. PHQ-4 = Patient Health Questionnaire-4; GAD-7 = Generalized Anxiety Disorder Scale; TMD = temporomandibular disorder

	Negative TMD (n = 151)	Positive TMD (n = 73)	P-value
Sex			
Male, n (%)	45 (29.8)	12 (16.4)	0.021*
Female, n (%)	106 (70.2)	61 (83.6)	
Age (years)			
<18, n (%)	2 (1.3)	0 (0)	0.763
18–39, n (%)	93 (61.6)	47 (64.4)	
40–59, n (%)	39 (25.8)	19 (26)	
>60, n (%)	17 (11.3)	7 (9.6)	
PHQ-4			
Normal, n (%)	77 (51)	18 (24.7)	0.001*
Mild, n (%)	48 (31.8)	37 (50.7)	
Moderate, n (%)	17 (11.3)	8 (11)	
Sever, n (%)	9 (6)	10 (13.7)	
GAD-7			
Minimal, n (%)	73 (48.3)	20 (27.4)	0.018*
Mild, n (%)	48 (31.8)	28 (38.4)	
Moderate, n (%)	17 (11.3)	15 (20.5)	
Sever, n (%)	13 (8.6)	10 (13.7)	

Results for Spearman correlations showed a significant positive relationship between TMD score and GAD-7 and PHQ-4 (p = <0.001). There was no significant relationship between GAD-7 and TMD among men. There was a significant relationship between TMD and PHQ-4 and GAD-7 among women. Both relationships were positive, as shown in Table 5.

**Table 5:** Spearman correlation coefficients between TMD score and PHQ-4 and GAD-7. PHQ-4 = Patient Health Questionnaire-4; GAD-7 = Generalized Anxiety Disorder Scale; TMD = temporomandibular disorder

	PHQ-4 and TMD		GAD-7 and TMD	
	r	P-value	r	P-value
Male	0.349	0.008	0.202	0.132
Female	0.291	>0.001	0.281	>0.001
Total	0.323	>0.001	0.275	>0.001

## Discussion

In this cross-sectional study, we investigated the relationships between TMDs and psychological distress in Saudi Arabia’s Al-Qassim region. The data supported our hypotheses. Even in this small study sample, people with TMD symptoms had a higher prevalence of depression and anxiety than those without psychological distress symptoms.

The number of female participants was higher than male participants, with 167 (74.6%) participants being female and 57 (25.4%) being male. Similar findings were reported in another study in Thailand [1]. Pain-related TMD was present in 73 (32.6%) respondents, whereas negative pain-related TMD was noted in 151 (67.4%) respondents. More than half of the participants (133; 59.4%) reported having at least one symptom of pain-related TMDs, which is high compared with other studies conducted in Thailand, Saudi Arabia, Riyadh, and China using the same TMD pain screener [1-3].

The rate of women with TMDs, 61 (83.6%), was higher than that of men, 12 (16.4%). This female predominance was observed in other studies in Thailand, China, and Sweden [1,3,4].

Two additional questions were asked of patients with TMDs regarding difficulties sleeping and whether they were seeking treatment. Overall, 37% of TMD patients had trouble sleeping. A study from China reported that the prevalence of moderate-to-severe sleep disturbance and psychological distress was significantly higher in myofascial pain patients than in nonmyofascial pain patients [4]. Additionally, 90.4% of TMD-positive patients had not sought medical or dental treatment. This finding indicates that either the population is not fully aware of the condition or there is a lack of care for health in general. Another possible factor is that the diagnosis of TMD is a challenge to all clinicians because of the complexity of the anatomical area and the difficulty of locating orofacial pain. According to a study conducted in the United States, patients with TMDs see more than three doctors before being referred to a tertiary care center. It was also discovered that a significant majority of those providers are physicians rather than dentists, and patients reported having a variety of diagnostic tests performed. According to a previous study, patients reported receiving a variety of diagnoses for their disease [17].

Most of the participants were aged 18 to 39 years (140; 62.5%), which was similar to studies in Saudi Arabia, Riyadh, Sweden, and Brazil [2,5,6]. However, in Thailand, middle-aged adults were the majority, with a mean age of 43.3 ± 14.9 years. An age difference in prevalence was also found in a study in Taiwan, which showed that the mean age for those with TMD was 52.31 ± 17 years [18]. According to epidemiological research conducted in the United States, Sweden, and Greece, the frequency of TMD symptoms decreases with age [7,8,19].

The prevalence of depression and anxiety varies among populations with different cultural and social environments. Our study showed an association between TMD pain and depression. This is in line with other studies that found an association between depression and TMD symptoms [20,21]. Other studies have identified a weak significant correlation between depression and TMD symptoms [1,22]. According to this study and other studies from Singapore, Serbia, China, and the Netherlands, patients with pain-related TMDs are more depressed than controls [4,21,23,24]. All individuals with pain-related TMDs may show signs of depression even if the precise relationship between TMDs and depression remains unknown. Further research is necessary to determine whether pain-related TMDs are present in patients with depression.

Anxiety is a psychological element that influences TMD discomfort. Anxiety was substantially linked with TMD pain, as assessed by the GAD-7. Other investigations conducted in Thailand and the Netherlands revealed this [1,24], and other studies using dissimilar metrics [2,4,21,23]. Additionally, compared to controls, patients with pain-related TMDs displayed significantly greater levels of anxiety, which is consistent with other research from Thailand and the Netherlands using a similar anxiety assessment scale [1,24]. Other research, including those in Singapore, China, and Sweden, used various methodologies [4,10,21].

However, a retrospective investigation revealed that, although the proportion of chronic TMD patients with severe anxiety increased as their level of impairment increased, anxiety was less significant than depression in the development of chronic TMD [9]. Therefore, it is unclear how depression and anxiety affect various aspects of TMDs [9,11,24]. It can be difficult to determine which of these two illnesses is more important for the emergence of TMDs because of their correlation.

Limitations

The data collected were based on participant self-recall and reporting of TMD symptoms, which can be considered a limitation. Therefore, there might be a significant discrepancy between self-reported symptoms and clinically documented symptoms. Future studies should include a clinical assessment of dental, muscular, and TMJ pathology. Additionally, a larger study sample would help reveal further findings.

## Conclusions

Residents of the Al-Qassim region had a significant prevalence of pain-related TMDs. Regardless of gender, significant favorable correlations between PHQ-4 and GAD-7 scores and symptoms of pain-related TMDs were noted. Patients who experienced elevated levels of anxiety and depression had significantly more pain-related TMD symptoms. These findings imply a link between psychological distress and TMD symptoms.
